# Discovery of Ro 41‐0960, a Non‐Steroidal Inhibitor of the Na^+^/K^+^‐ATPase

**DOI:** 10.1002/cmdc.70442

**Published:** 2026-08-28

**Authors:** Carlos Cruz‐Cortés, Jaroslava Šeflová, Guadalupe Guerrero‐Serna, Eric N. Jimenez‐Vazquez, Justus Anumonwo, L. Michel Espinoza‐Fonseca

**Affiliations:** ^1^ Department of Internal Medicine Division of Cardiovascular Medicine University of Michigan Ann Arbor Michigan USA; ^2^ Center for Arrhythmia Research University of Michigan Ann Arbor Michigan USA; ^3^ Department of Cell and Molecular Physiology Loyola University Chicago Maywood Illinois USA; ^4^ Department of Health and Biomedical Sciences University of Texas Rio Grande Valley Brownsville Texas USA; ^5^ Frankel Cardiovascular Center Department of Internal Medicine Division of Cardiovascular Medicine University of Michigan Ann Arbor Michigan USA; ^6^ Department of Pharmacology University of Michigan Ann Arbor Michigan USA

**Keywords:** allosteric regulation, enzyme inhibition, Na^+^/K^+^‐ATPase, Na‐K ATPase inhibitor, pharmacology

## Abstract

The Na^+^/K^+^‐ATPase (NKA) is a central regulator of cardiac ion homeostasis and a validated target for heart failure. Yet, cardiotonic steroids used in clinical practice are limited by a narrow therapeutic window and pro‐arrhythmic effects. Here, we identify Ro 41‐0960 as a non‐steroidal inhibitor of NKA with a distinct mechanistic profile. The compound inhibits NKA activity with IC_50_ values of 17.9 ± 1.1 µM for purified enzyme and 10.3 ± 1.1 µM in microsomal preparations. ATP‐dependent activity measurements revealed a non‐monotonic response in which inhibition was most pronounced at ATP concentrations below 4 mM and diminished at the highest ATP concentrations tested. Docking and molecular dynamics simulations suggest that Ro 41‐0960 can access both the cardiotonic steroid‐binding pocket and the nucleotide‐binding site; however, the ATP‐dependence data are not consistent with a simple ATP‐competitive mechanism. Furthermore, the compound shows only weak inhibition of SERCA (IC_50_ > 100 µM) and does not alter electrophysiological parameters in human iPSC‐derived cardiomyocytes at concentrations producing near‐maximal NKA inhibition, providing initial evidence of a favorable cardiac safety profile. Together, these findings identify Ro 41‐0960 as an NKA inhibitor with a distinct chemical scaffold and provide a framework for the development of non‐steroidal NKA modulators.

## Graphical abstract

Ro 41‐0960 is a non‐steroidal Na^+^/K^+^‐ATPase inhibitor with low micromolar potency and a mixed inhibitory mechanism distinct from classical cardiotonic steroids, without acute pro‐arrhythmic effects in human cardiomyocytes.

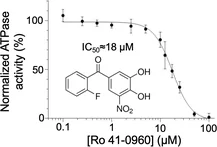

## 1 Introduction

Heart failure (HF) remains a major global health problem and is a leading cause of morbidity and mortality in the United States, accounting for 1 in 9 deaths and imposing an annual economic burden exceeding $32 billion [cdc.gov]. Despite advances in device‐based and pharmacological therapies, the prognosis for HF remains poor, with a 5‐year mortality rate approaching 50%. While heart transplantation offers a potential cure, donor organ availability limits its utility to a small fraction of patients. Current evidence emphasizes the unfavorable outcomes of HF in patients under optimized treatment, and symptom‐based HF treatments have shown only modest improvement in mortality [1, 2]. Despite major advances in guideline‐directed medical therapy, HF remains associated with substantial morbidity and mortality, highlighting the need for therapeutics that directly target the molecular mechanisms responsible for impaired cardiac contractility [3].

Congestive HF, which affects more than 5 million people in the United States and is particularly prevalent in individuals over 65 years of age, is characterized by impaired left ventricular filling or reduced ejection of blood to the systemic circulation [2]. A well‐established positive inotropic strategy for the treatment of HF is inhibition of the Na^+^/K^+^‐ATPase (NKA) by cardiotonic steroids (CTSs) [4]. This approach exploits the physiological coupling between sodium and calcium gradients via the sodium–calcium exchanger (NCX): inhibition of NKA reduces the transmembrane Na^+^ gradient, thereby decreasing Ca^2+^ extrusion through NCX and increasing intracellular Ca^2+^ levels, resulting in enhanced force of contraction (positive inotropy) [5]. This mechanism underlies the therapeutic action of CTSs, which exert their effects through inhibition of NKA [6]. Despite their long‐standing clinical use, CTSs suffer from major limitations that restrict their therapeutic value. Most notably, they exhibit a narrow therapeutic window [7], in which plasma concentrations only slightly above the effective range are associated with toxicity, including life‐threatening ventricular arrhythmias [8]. In addition, while these agents can provide symptomatic and inotropic benefits in selected clinical settings, their impact on long‐term survival and overall clinical outcomes has been limited compared with newer HF therapies [9]. These limitations highlight the need for next‐generation NKA modulators that retain the beneficial inotropic effects of CTSs while minimizing proarrhythmic liability and expanding the pharmacological space for safer mechanistically distinct compounds.

In our previous work, we identified the synthetic compound Ro 41‐0960 as a membrane‐active small molecule that modulates Ca^2+^ handling in human iPSC‐derived cardiomyocytes [10]. Notably, Ro 41‐0960 exerts its effects at the cellular level in a manner not consistent with canonical SERCA activation [11, 12], suggesting involvement of alternative ion transport mechanisms. We therefore hypothesized that Ro 41‐0960 may engage membrane‐embedded transporters involved in cardiac excitation–contraction coupling. Given the central role of NKA in regulating intracellular Na^+^ and Ca^2+^ homeostasis [5], these observations provided a rationale to examine the effects of Ro 41‐0960 on NKA. Here, we identify Ro 41‐0960 as a non‐steroidal inhibitor of NKA with a complex ATP‐dependent inhibitory profile that is not readily explained by a simple classical ATP‐competitive mechanism. Importantly, Ro 41‐0960 does not induce pro‐arrhythmic effects in human iPSC‐derived cardiomyocytes at concentrations up to 50 µM. Together, these findings establish a non‐steroidal scaffold for targeting NKA and expand the chemical space of small‐molecule inhibitors directed at this pump.

## 2 Results and Discussion

### 2.1 Identification of Ro 41‐0960 as a Low Micromolar Inhibitor of NKA

**FIGURE 1:**
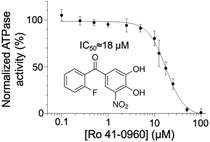
Concentration‐dependent inhibition of NKA by Ro 41‐0960. ATPase activity of purified NKA (α1 isoform) is shown as a function of Ro 41‐0960 concentration. Enzyme activity is normalized to control conditions in the absence of compound and 3 mM ATP. Data were fitted using a four‐parameter nonlinear concentration–response model with a variable slope, yielding an IC_50_ value of 17.9 ± 1.1 µM. The chemical structure of Ro 41‐0960 is shown. Data represent mean ± SEM from independent experiments (*N* = 3).

Ro 41‐0960 inhibited the activity of purified NKA in a clear concentration‐dependent manner, with minimal effects at submicromolar concentrations and a progressive reduction in activity as concentration increased (Figure 1). Analysis of the concentration–response relationship yielded an IC_50_ of 17.9 ± 1.1 µM, with substantial inhibition observed at 20–50 µM and near‐complete inhibition at 50–100 µM. This apparent IC_50_ was determined under the defined assay conditions used here (Experimental Section, NKA ATPase Activity Assays), including 3 mM ATP, and should not be interpreted as an intrinsic ATP‐independent inhibition constant. The sigmoidal inhibition profile is consistent with a defined interaction with the enzyme rather than nonspecific loss of activity at high compound concentrations.

**FIGURE 2:**
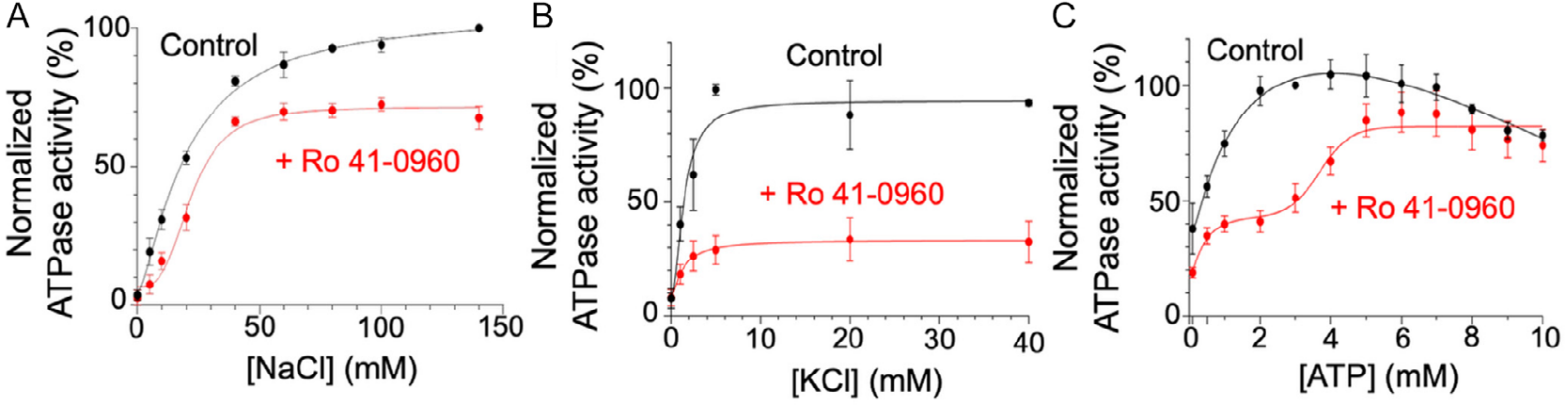
Effects of Ro 41‐0960 on Na^+^‐, K^+^‐, and ATP‐dependent activity of purified NKA. (A) ATPase activity as a function of NaCl concentration, measured using the coupled enzyme assay. (B) ATPase activity as a function of KCl concentration, measured using the direct phosphate‐release assay. (C) ATPase activity as a function of ATP concentration, measured using the coupled enzyme assay. In all panels, enzyme activity in the absence (control, black) and presence of Ro 41‐0960 (red) is shown. In all cases, Ro 41‐0960 was evaluated at a concentration of 18 µM. The substrate‐dependence experiments shown in each panel were performed as independent experimental series and normalized independently to the corresponding control reference activity. ATP‐dependent activities in panel C were normalized to the maximal control activity observed at 3–4 mM ATP. Data in panels A and B were fitted to a nonlinear allosteric sigmoidal model. Data in panel C were fitted to a nonlinear bell‐shaped model for the control condition and a nonlinear biphasic model in the presence of Ro 41‐0960. Data represent mean ± SEM from independent experiments (*N* = 3).

To further gain insight into the mechanism of inhibition, we examined NKA activity as a function of Na^+^ and K^+^ concentrations in the presence and absence of Ro 41‐0960. Ro 41‐0960 was used at the corresponding IC_50_ concentration estimated from the concentration–response curve (Figure 1). Across all conditions, the presence of the compound resulted in a pronounced reduction in maximal ATPase activity (Figure 2A,B). In both Na^+^‐ and K^+^‐dependent measurements, Ro 41‐0960 decreased activity across the entire concentration range without producing a substantial rightward shift in the apparent activation curves. Consistent with this observation, the apparent K_0.5_ values for Na^+^ were 19.3 ± 2.1 and 20.0 ± 1.7 mM in the absence and presence of Ro 41‐0960, respectively, whereas the apparent K_0.5_ values for K^+^ were 1.3 ± 0.3 and 0.8 ± 0.7 mM under the two conditions. These differences were not statistically significant (*t*‐test, *p* = 0.81 for Na^+^ and *p* = 0.59 for K^+^). This behavior is inconsistent with simple competitive displacement of either ion, but suggests that Ro 41‐0960 perturbs the catalytic cycle in a manner that reduces overall turnover while largely preserving ion‐dependent activation.

A more complex behavior was observed in ATP‐dependent measurements (Figure 2C) in presence or absence of 18 µM Ro 41‐0960. Under control conditions, NKA activity exhibited a non‐monotonic dependence on ATP concentration, reaching maximal activity at approximately 3–4 mM ATP and decreasing at higher ATP concentrations, consistent with previous observations using a direct phosphate‐release assay [13]. Ro 41‐0960 reduced NKA activity primarily over ATP concentrations below 4 mM. At the highest ATP concentrations tested (8–10 mM), however, the control and Ro 41‐0960‐treated activities converged. Thus, the inhibitory effect of Ro 41‐0960 is ATP‐dependent and is diminished at elevated ATP concentrations. Although this behavior is compatible with a contribution from ATP‐dependent steps, the complex, non‐monotonic ATP dependence and absence of a simple rightward displacement of the control curve do not unequivocally allow assignment of a classical ATP‐competitive mechanism based on these data alone.

### 2.2 Biochemical Characterization of Ro 41‐0960 Inhibition of NKA

**FIGURE 3:**
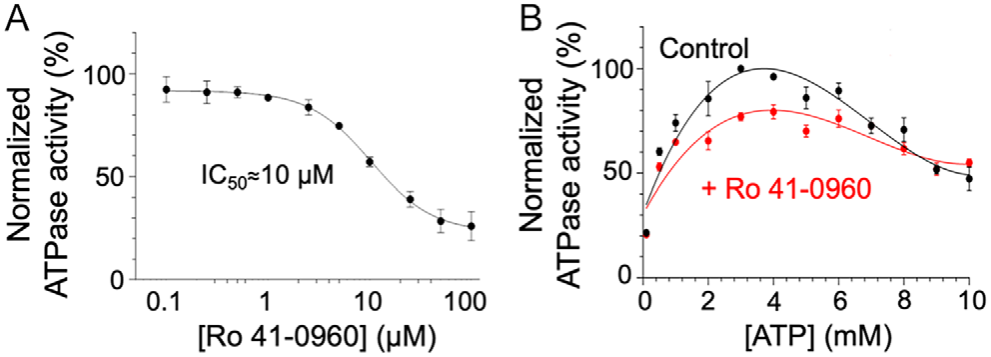
Inhibition of NKA by Ro 41‐0960 in microsomal preparations. (A) Concentration‐dependent inhibition of NKA activity measured using the coupled enzyme assay, yielding an IC_50_ of 10.3 ± 1.1 µM at 3 mM ATP. (B) ATP dependence of NKA activity measured using the coupled enzyme assay in the absence (control, black) and presence of 18 µM Ro 41‐0960 (red). ATP‐dependent activities were normalized to the maximal control activity observed at 3–4 mM ATP. Data in panel A were fitted using a four‐parameter nonlinear concentration–response model with a variable slope. Data in panel B were fitted to a nonlinear bell‐shaped model. Data represent mean ± SEM from independent experiments (*N* = 3).

To further resolve the relative contribution of orthosteric versus allosteric inhibition, we next evaluated the effect of Ro 41‐0960 in intact microsomal preparations, which preserve the native membrane environment and ionic conditions of NKA. Ro 41‐0960 inhibited NKA activity in microsomes with comparable apparent potency under the defined assay conditions used here (IC_50_ = 10.3 ± 1.1 µM at 3 mM ATP; Figure 3A), indicating that its inhibitory effect is maintained in a physiologically relevant lipid environment. Notably, residual ouabain‐sensitive ATPase activity persisted at high Ro 41‐0960 concentrations in microsomal preparations (Figure 3A), indicating partial inhibition of NKA catalytic turnover in the native membrane environment rather than complete suppression of ATP hydrolysis.

To assess whether ATP‐dependent steps contribute to the inhibitory mechanism, we examined NKA activity in microsomal preparations as a function of ATP concentration in the presence and absence of 10 µM Ro 41‐0960, corresponding to the IC_50_ determined in Figure 3A. Under control conditions, NKA activity exhibited a non‐monotonic dependence on ATP concentration (Figure 3B), reaching maximal activity at approximately 3–4 mM ATP and subsequently decreasing at higher ATP concentrations. Similar non‐monotonic ATP‐dependent behavior has been reported previously for NKA using direct phosphate‐release measurements [13]. ATP‐dependent activity values were normalized to the maximal control activity observed at 3–4 mM ATP. Ro 41‐0960 reduced NKA activity primarily over the low‐to‐intermediate ATP concentration range, whereas the control and inhibitor‐treated curves converged at 8–10 mM ATP. Thus, the inhibitory effect of Ro 41‐0960 is ATP‐dependent and is diminished at the highest ATP concentrations tested. However, convergence of the curves at elevated ATP concentrations alone does not establish a classical ATP‐competitive mechanism. The control ATP‐dependence is inherently non‐monotonic, and the Ro 41‐0960‐treated curve displays a similarly complex ATP dependence rather than a simple rightward displacement of the control curve. Taken together, these findings indicate that the apparent inhibitory potency of Ro 41‐0960 depends on ATP concentration under the experimental conditions used here, but they do not support a classical ATP‐competitive mechanism.

### 2.3 Ro 41‐0960 Selectively Inhibits NKA Over the Homologous SERCA Pump

The nucleotide‐binding domain of P‐type ATPases is highly conserved across family members, including NKA and SERCA, reflecting a shared catalytic mechanism centered on ATP binding and phosphorylation [14, 15]. As a result, inhibitors that act through direct competition at the nucleotide‐binding site would be expected to exhibit limited selectivity between these homologous transporters. To test this possibility, we evaluated the effects of Ro 41‐0960 on the activity of the homologous cardiac calcium pump SERCA under comparable membrane‐associated conditions. Because SERCA activity was measured in sarcoplasmic reticulum microsomes, comparison with NKA inhibition was performed using microsomal preparations to preserve a similar membrane environment. SERCA provides a stringent test of this hypothesis, as it shares a highly conserved catalytic core and ATP‐binding architecture with NKA [14, 15] but lacks an equivalent CTS binding pocket, consistent with structural analyses of ligand‐binding sites in SERCA [16].

**FIGURE 4:**
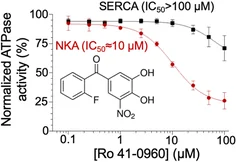
Selective inhibition of NKA over cardiac SERCA by Ro 41‐0960 in microsomal preparations. Concentration‐dependent inhibition of NKA (red) and SERCA (black) ATPase activity measured using coupled enzyme assays under the respective assay conditions described in the experimental section. The NKA concentration‐response dataset is reproduced from Figure 3A to facilitate direct comparison with the SERCA concentration‐response data. Ro 41‐0960 inhibits NKA with an IC_50_ of 10.3 ± 1.1 µM at 3 mM ATP, SERCA activity is only weakly inhibited at substantially higher concentrations (IC_50_ > 100 µM). In both cases, data were fitted using a four‐parameter nonlinear concentration–response model with a variable slope. Data represent mean ± SEM from independent experiments (*N* = 3).

In contrast to the pronounced inhibition observed for NKA, Ro 41‐0960 exhibited only weak inhibition of SERCA (IC_50_ > 100 µM), with measurable effects detected only at substantially higher compound concentrations (Figure 4). Notably, at concentrations corresponding to the IC_50_ for NKA inhibition (i.e., ~10 µM), SERCA activity remained largely unaffected, indicating a marked difference in sensitivity between the two enzymes (Figure 4). This differential sensitivity suggests that the inhibitory effect of Ro 41‐0960 on NKA is not solely driven by interactions with the conserved ATP‐binding site shared by NKA and SERCA. However, the weak inhibition of SERCA observed at higher compound concentrations is consistent with the possibility that interactions with conserved structural features, including the nucleotide‐binding site, contribute to the inhibitory activity of Ro 41‐0960. The substantially greater sensitivity of NKA suggests that additional interactions specific to NKA may contribute to its inhibitory potency.

### 2.4 Molecular Simulations Suggest Dual‐Site Binding of Ro 41‐0960 to NKA

**FIGURE 5:**
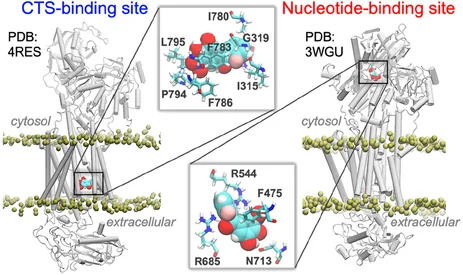
Predicted binding modes of Ro 41‐0960 in CTS‐ and nucleotide‐binding sites of NKA. Representative docking poses of Ro 41‐0960 within the CTS‐binding pocket (left; PDB: 4RES) and the nucleotide‐binding site (right; PDB: 3WGU) of pig NKA (α1 isoform). Insets show close‐up views of predicted ligand positioning and surrounding residues in each binding site. These models illustrate plausible static binding orientations used as starting configurations for subsequent MD simulations. In all panels, NKA is shown in cartoon representation, Ro 41‐0960 is shown as spheres, and interacting residues are depicted as sticks. The approximate position of the lipid bilayer is indicated by tan spheres. Extracellular and cytosolic orientations are indicated for structural reference.

The biochemical data indicate that the inhibitory effect of Ro 41‐0960 depends on ATP concentration but cannot be assigned to a simple classical ATP‐competitive mechanism. To explore potential binding regions, we performed molecular docking followed by atomistic molecular dynamics simulations of Ro 41‐0960 in complex with NKA. The CTS‐binding pocket and the nucleotide‐binding site were evaluated as candidate sites because they represent well‐characterized, chemically accessible ligand‐binding cavities in NKA capable of accommodating small molecules. Each system was simulated on the microsecond timescale to assess the persistence of ligand–protein interactions under dynamic conditions. The purpose of this analysis was to identify plausible binding modes for Ro 41‐0960 rather than to imply that the compound reproduces the functional mechanism of classical CTS. The absence of a detectable change in apparent K^+^ dependence suggests that the biochemical behavior of Ro 41‐0960 may differ from that of classical CTS, although these activity measurements do not directly assess reciprocal interactions between K^+^ and ligand binding. Docking calculations identified plausible poses within both the CTS‐binding pocket and the nucleotide‐binding pocket (Figure 5). The predicted poses allow favorable interactions with surrounding residues, including hydrophobic contacts within the transmembrane cavity and polar interactions within the nucleotide‐binding site.

**FIGURE 6:**
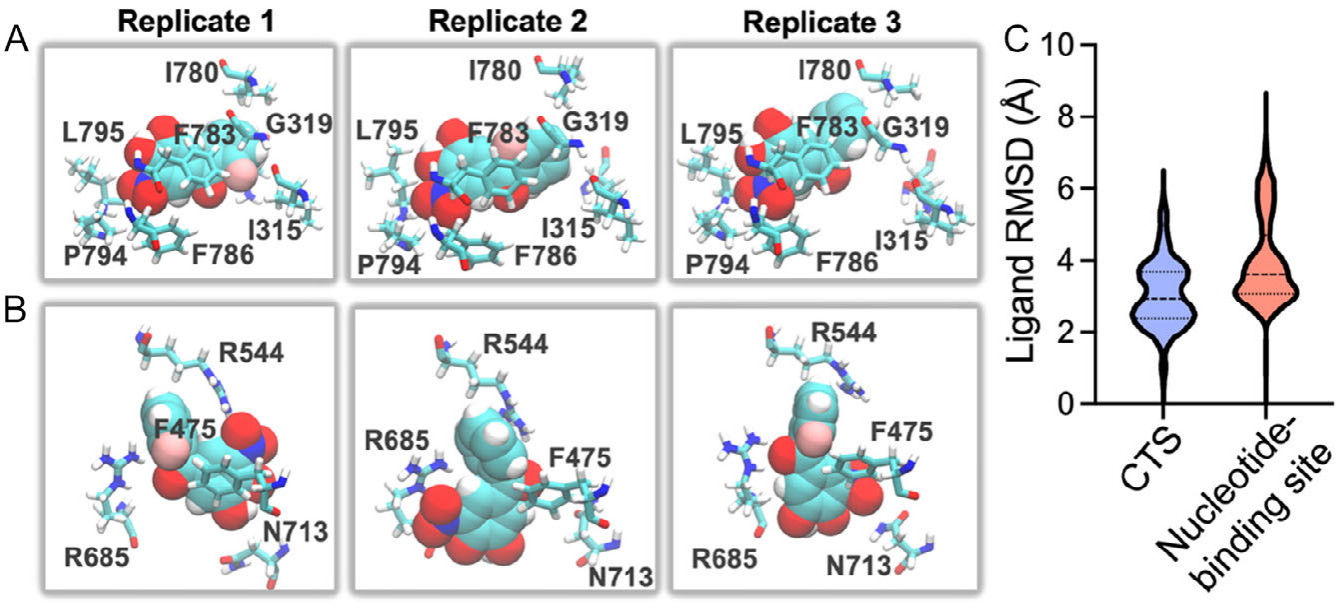
Dynamic behavior of Ro 41‐0960 in CTS‐ and nucleotide‐binding sites during MD simulations. Ligand positional behavior across independent MD replicates in the (A) CTS‐binding pocket and (B) nucleotide‐binding site. In the CTS‐binding pocket, Ro 41‐0960 remains more positionally confined near residues contributing aromatic and hydrophobic contacts, including Phe783, Phe786, Ile780, Leu795, and Pro794. In the nucleotide‐binding site, Ro 41‐0960 samples broader poses near residues including Phe475, Arg544, Arg685, and Asn713. (C) Distribution of ligand RMSD values relative to the starting docking pose, showing lower and more narrowly distributed RMSD values in the CTS‐binding pocket than in the nucleotide‐binding site, supporting greater positional stability of Ro 41‐0960 within the CTS‐binding pocket.

Molecular dynamics (MD) simulations showed that in the CTS‐binding pocket, Ro 41‐0960 remained closely associated with the initial docking pose and exhibited consistent positional confinement across MD replicates (Figure 6A). This behavior was supported by persistent aromatic and hydrophobic contacts with residues lining the pocket, including Phe783, Phe786, Ile780, Leu795, and Pro794. In particular, the nitrocatechol moiety of the compound maintained stable aromatic interactions with Phe783, a key residue involved in stabilizing CTSs within this site [17]. In contrast, Ro 41‐0960 exhibited greater positional variability in the nucleotide‐binding site and deviated more substantially from the initial docking pose across MD replicates (Figure 6B). Although the ligand remained associated with the binding region, interactions with residues in the nucleotide‐binding site, including Phe475, Arg544, Arg685, and Asn713, were less spatially constrained and consistent with broader conformational sampling. Consistent with these observations, Ro 41‐0960 exhibited lower and more narrowly distributed ligand RMSD values in the CTS‐binding pocket than in the nucleotide‐binding site, with median RMSD values of ~2.7 and ~3.7 Å, respectively (Figure 6C).

Taken together, the simulations indicate that Ro 41‐0960 can adopt plausible binding poses in both the CTS‐binding pocket and the nucleotide‐binding region, with greater positional confinement observed in the CTS pocket during the simulations. These computational results are hypothesis‐generating and do not establish the functional binding site or a CTS‐like mechanism of inhibition. The ATP‐dependence experiments indicate that inhibition is diminished at the highest ATP concentrations tested, indicating an ATP‐dependent component to the inhibitory behavior. At the same time, the complex non‐monotonic ATP dependence and absence of a simple rightward displacement of the ATP‐dependence curve do not permit assignment of a classical ATP‐competitive mechanism. Thus, the biochemical and computational data together support the possibility of multiple interactions or conformational effects, but additional studies will be required to define the binding site and molecular mechanism of Ro 41‐0960 inhibition.

### 2.5 Ro 41‐0960 Does Not Induce Pro‐Arrhythmic Events in Human Cardiomyocytes

**FIGURE 7:**
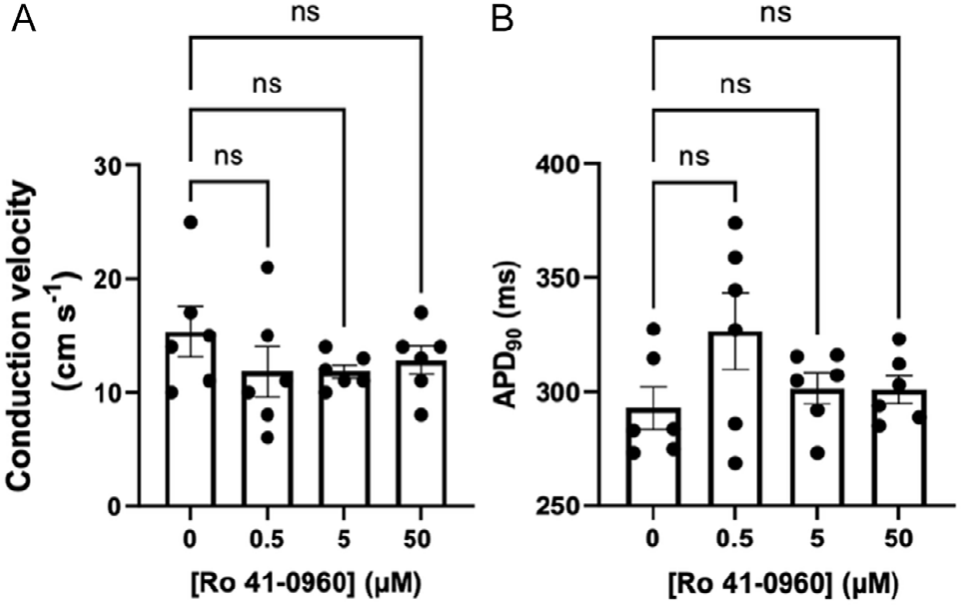
Ro 41‐0960 does not alter impulse propagation and APD_90_ in human iPSC‐derived cardiomyocytes. (A) Conduction velocity and (B) action potential duration at 90% repolarization (APD_90_) measured in spontaneously beating human iPSC‐derived cardiomyocytes across increasing concentrations of Ro 41‐0960 (0–50 µM). Significance was evaluated using a two‐way ANOVA followed by Dunnett's post‐hoc test. No significant differences were observed relative to control conditions (ns, not significant). Data are presented as mean ± SEM with individual data points shown (*N* = 6).

Finally, we examined the potential for drug‐induced cardiac toxicity, an important consideration in drug development, as compounds with beneficial pharmacological effects could elicit pro‐arrhythmic side effects [18, 19]. To evaluate the pro‐arrhythmic potential of Ro 41‐0960, we performed cardiotoxicity screening using high‐resolution optical mapping with a voltage‐sensitive dye to quantify conduction velocity and action potential duration at 90% repolarization (APD_90_) in spontaneously beating human iPSC‐derived cardiomyocytes. Conduction velocity remained unchanged across the tested concentration range (0.5–50 µM), indicating that the compound does not impair electrical impulse propagation through the cardiomyocyte syncytium (Figure 7A). Similarly, APD_90_ was not significantly altered, with no evidence of prolongation that would suggest increased arrhythmogenic risk (Figure 7B). No statistically significant changes in conduction velocity or APD_90_ were observed at any concentration tested, including at 50 µM, which produces near‐complete inhibition of NKA. These findings are important because alterations in conduction velocity and/or APD_90_ are commonly associated with pro‐arrhythmic mechanisms, including re‐entrant excitation and early or delayed afterdepolarizations [19, 21]. This interpretation is consistent with our observation that neither early nor delayed afterdepolarization events were detected at any concentration of the compound. While these results do not exclude the possibility of arrhythmogenic effects under stress conditions or prolonged exposure, they provide initial evidence that Ro 41‐0960 does not exhibit acute pro‐arrhythmic signatures at concentrations relevant to NKA inhibition. Together, these findings support a favorable electrophysiological profile and further distinguish Ro 41‐0960 from traditional NKA inhibitors.

## 3 Conclusion

In summary, we identify Ro 41‐0960 as a non‐steroidal inhibitor of the Na^+^/K^+^‐ATPase with a complex ATP‐dependent inhibitory profile. Biochemical analyses show that inhibition is most pronounced at low‐to‐intermediate ATP concentrations and diminishes at the highest ATP concentrations tested. However, the non‐monotonic ATP dependence and absence of a rightward displacement of the ATP‐dependence curve do not permit assignment of a simple ATP‐competitive mechanism. Complementary docking and molecular dynamics simulations identify plausible interactions within both the CTS‐binding pocket and the nucleotide‐binding region, with greater positional stability predicted in the CTS pocket. These computational findings are hypothesis‐generating and do not establish the functional binding site of Ro 41‐0960. Importantly, Ro 41‐0960 showed no acute pro‐arrhythmic effects in human iPSC‐derived cardiomyocytes at concentrations relevant to NKA inhibition, providing initial evidence of a favorable cardiac safety profile. Collectively, these findings expand the chemical space of non‐steroidal small‐molecule inhibitors targeting NKA and provide a foundation for further mechanistic and medicinal chemistry studies.

## 4 Experimental Section

### 4.1 Chemicals

All chemicals used in this study were purchased at reagent quality (purity > 95% by HPLC) and purchased from Sigma (St. Louis, MO, USA) and Thermo‐Fisher Scientific (Waltham, MA, USA) unless otherwise indicated.

### 4.2 NKA Isolation

NKA (α1 isoform) was isolated from porcine kidney outer medulla using established protocols [22, 24], with some modifications. Briefly, fresh porcine kidneys were obtained post‐mortem from the University of Michigan Unit for Laboratory Animal Medicine. The outer medulla was dissected, mixed with ice‐cold buffer composed of 250 mM sucrose, 30 mM L‐histidine (pH 7.3) in 1:1 (tissue:buffer) ratio, and incubated in cold room overnight. The tissue was minced and homogenized in ice‐cold buffer containing 250 mM sucrose, 20 mM imidazole, and 1 mM EDTA (pH 7.4) using a glass‐Teflon homogenizer. The homogenate was subjected to differential centrifugation at 3700 g for 20 min at 4 °C, followed by centrifugation of the supernatant at 7400 g for 20 min to remove cell debris. The supernatant was subjected to additional centrifugation at 38,000 g for 40 min to pellet the microsomal membranes. The microsomal membranes were aliquoted, frozen in liquid nitrogen, and stored at −80 °C. The identity of the α1 isoform was confirmed by Western blot analysis.

### 4.3 NKA Purification

Plasma membrane microsomes containing NKA were resuspended and solubilized in 0.1% (w/v) sodium dodecyl sulfate (SDS) in a buffer containing 250 mM sucrose, 0.9 mM EDTA, and 20 mM L‐histidine (pH 7.0). The solubilized membranes were subjected to density‐gradient ultracentrifugation to isolate the purified enzyme. The resulting NKA preparation was aliquoted, flash‐frozen in liquid nitrogen, and stored at −80 °C in stabilization buffer containing 250 mM sucrose, 0.9 mM EDTA, and 20 mM L‐histidine (pH 7.0), supplemented with a trace amount of SDS to prevent aggregation. Protein concentration was determined by the Bradford assay using bovine serum albumin as a standard. Enzyme purity was verified by SDS‐PAGE followed by Coomassie staining. Functional activity was confirmed by measuring ouabain (OUA)‐sensitive ATP hydrolysis prior to use in subsequent experiments.

### 4.4 NKA ATPase Activity Assays

We tested the activity Ro 41‐0960 by measuring the ATPase activity of sensitive to 500 μM OUA (in μmol min^−1^ mg^−1^) from the decrease in absorbance of NADH at 340 nm at 37 °C in a 96‐well format using a Synergy H1 microplate reader (BioTek, Winooski, VT). Each well contained a 200 μL final volume of assay buffer containing NKA buffer (30 mM MOPS, 130 mM NaCl, 20 mM KCl, and 4 mM MgCl2, pH = 7.4), 5U lactate dehydrogenase, 5U pyruvate dehydrogenase, 1 mM phosphoenolpyruvate, 3 mM ATP, 0.2 mM NADH, and 0.8 μg of the purified NKA suspension. The enzyme preparation was preincubated with Ro 41‐0960 for 30 min before the reaction was initiated by ATP addition. Following ATP addition, absorbance was monitored continuously at 1‐min intervals for 60 min. To assess the ATPase activity at different concentrations of Na^+^ or ATP, we prepared serial dilutions of NaCl (0–140 mM) and ATP (0.1–10 mM). Because pyruvate kinase requires K^+^ as a cofactor, K^+^‐dependent ATPase activity was measured using a direct phosphate‐release assay to avoid potential interference from the coupling enzymes. ATP hydrolysis was quantified by measuring inorganic phosphate (P_i_) released during the reaction using a modified colorimetric assay based on Bartolommei et al. [25]. The assay buffer contained: 130 mM NaCl, 30 mM MOPS, 4 mM MgCl_2_, and KCl at 0, 1, 2.5, 5, 20, or 40 mM. Samples containing 20 μg total protein were incubated for 30 min at 37 °C with or without Ro 41‐0960, and ATP hydrolysis was initiated by adding 1 mM ATP. The use of 1 mM ATP followed the previously validated conditions of Bartolommei et al. [25] and was selected to assess K^+^‐dependent activation and apparent K^+^ affinity rather than maximal ATPase activity. ATP hydrolysis was initiated immediately upon ATP addition. Aliquots were collected every minute during the initial linear phase of the reaction (0–5 min), and ATPase activity was determined from the linear rate of phosphate production over this interval. Each aliquot was immediately mixed with coloring reagent containing 2.5 M H_2_SO_4_, 300 mM ascorbic acid, 4 mM potassium antimony(III)tartrate, and 24 mM ammonium heptamolybdate. After incubation for 10 min at room temperature, absorbance was measured at 850 nm using a Synergy H1 microplate reader (BioTek, Winooski, VT). Ouabain‐sensitive ATPase activity was calculated by subtracting the residual activity measured in the presence of 500 μM ouabain. P_i_ release was quantified using a phosphate standard curve and expressed as nmol P_i_ released. In all cases, the residual activity in the presence of OUA was subtracted from total ATPase activity to determine the ouabain‐sensitive NKA ATPase activity. Concentration‐response and substrate‐dependence experiments were performed as independent experimental series using separate enzyme preparations and independently prepared reaction series. Data within each experimental series were normalized independently to the corresponding control reference activity. For the ATP‐dependence experiments shown in Figures 2C and 3B, activities were normalized to the maximal control activity observed at 3–4 mM ATP. Accordingly, normalized activities displayed in separate panels should not be interpreted as replicate determinations of fractional inhibition under identical experimental conditions. Representative absolute ATPase activities of the enzyme preparations used in this study are provided in Table S1. Data were analyzed by nonlinear regression using GraphPad Prism 10 (GraphPad Software, Boston, MA, USA). Concentration–response data were fitted using a four‐parameter logistic model with a variable slope to determine IC_50_ values. Ion‐dependence data were fitted using an allosteric sigmoidal model, whereas ATP‐dependence data were fitted using bell‐shaped or biphasic models, as appropriate. Data are presented as mean ± SEM from three independent experiments (*n* = 3).

### 4.5 SERCA Isolation

Cardiac SERCA (2a isoform) was isolated from porcine pig hearts obtained post‐euthanasia from the University of Michigan Unit for Laboratory Animal Medicine. Ventricle free walls were minced and homogenized with a cold buffer that contained 9.1 mM NaHCO_3_, 0.9 mM Na_2_CO_3,_ and a cocktail of protease inhibitors; the mixture was centrifuged at 6500 g for 30 min at 4 °C to remove debris. The supernatant was filtered, collected, and centrifuged at 14,000 *g* for 30 min at 4 °C. The collected filtrate was centrifuged at 47,000 g for 60 min at 4 °C. The pellet was resuspended in a solution containing 0.6 M KCl and 20 mM Tris (pH = 6.8). The suspension was centrifuged at 120,000 g for 60 min at 4 °C, and the pellet was resuspended in a solution containing 300 mM sucrose, 5 mM MOPS, and protease inhibitors (pH = 7.4). The protein concentration of the SR microsomal fraction was determined using the PierceTM Coomassie plus assay kit (Thermo‐Fisher Scientific, Waltham, MA). The microsomal membranes were aliquoted, frozen in liquid nitrogen, and stored at −80 °C.

### 4.6 SERCA ATPase Activity Assays

We tested the effects of Ro 41‐0960 on SERCA using activity assays as described previously [26]. Briefly, we measured the activity of Ca^2+^ ATPase in µmol min^−1^ mg^−1^ from the decrease in absorbance of NADH at 340 nm at 25 °C in a 96‐well format using a Synergy H1 (BioTek, Winooski, VT) microplate reader. Each well contained a 200 µL final volume of assay buffer containing SERCA buffer (50 mM MOPS, 100 mM KCl, 5 mM MgCl_2_, and 1 mM EGTA, pH = 7), 5U lactate dehydrogenase, 5U pyruvate dehydrogenase, 1 mM phosphoenolpyruvate, 5 mM ATP, 0.2 mM NADH, 2 µg of microsomal suspension, 2 µM of Ca^2+^ ionophore A23187, 1 µM free Ca^2+^, and Ro 41‐0960 concentrations of 0.1–100 µM. Prior to the ATPase assays, we incubated the small molecules for 30 min at 25 °C with the reaction mixture. ATP hydrolysis was initiated immediately upon ATP addition, and absorbance was monitored continuously at 1‐min intervals for 60 min. For comparative inhibition analyses, specific SERCA ATPase activities were normalized to untreated control preparations to account for variability across independent preparations. Data were analyzed by nonlinear regression using GraphPad Prism 10 (GraphPad Software, Boston, MA, USA). Concentration–response data were fitted using a four‐parameter logistic model with a variable slope. Absolute ATPase activities of SERCA preparations used in this study are summarized in Table S1.

### 4.7 Docking and Molecular Dynamics Simulations

Docking simulations were performed using CB‐Dock2 [27], which integrates cavity detection with AutoDock Vina 1.2 [28] for blind docking. The crystal structures of NKA in the E1 (PDB: 3WGU) [29] and E2 (PDB: 4RES) [30] conformations were used as receptor models. The 3D structure of Ro 41‐0960 was obtained from PubChem [31] (CID: 3495594) and energy‐minimized using the MMFF94s force field in Avogadro [32]. Blind docking was carried out across cavities identified by CB‐Dock2, and top‐ranked poses were selected for molecular dynamics (MD) simulations. Protein–ligand complexes were embedded in an asymmetric lipid bilayer mimicking the plasma membrane [33], composed of POPC:POPE (2:1) lipids in the extracellular leaflet and POPC:POPE:POPS (4:2:1) in the intracellular leaflet. Systems were constructed using CHARMM‐GUI [34, 35], solvated with OPC water, and neutralized with Na^+^, K^+^, and Cl^−^ ions to yield final Na^+^ and K^+^ concentrations of 130 and 20 mM, respectively. MD simulations were performed using AMBER24 with the ff14 SB force field for proteins [36] and LIPID21 for lipids [37]. After energy minimization and equilibration under standard NVT and NPT conditions, production simulations were carried out in the NPT ensemble at 37 °C and 1 bar using a Langevin thermostat and Monte Carlo barostat. Long‐range electrostatics were treated using periodic boundary conditions, and bonds involving hydrogen atoms were constrained using the SHAKE algorithm. Three independent 1‐µs simulations were performed for each system using Tesla V100 GPUs. Trajectory analysis was conducted using VMD [38].

### 4.8 Optical Mapping in Human iPSC‐Derived Cardiomyocytes

Cryopreserved human iPSC‐derived cardiomyocytes (iCell Cardiomyocytes2, FUJIFILM Cellular Dynamics, Madison, WI, USA) were thawed and plated onto custom 6‐well plates containing polydimethylsiloxane (PDMS) inserts (40D hardness, ~1000 kPa; Specialty Manufacturing, Inc., Saginaw, MI, USA). PDMS surfaces were sterilized and coated with Matrigel (500 μg/mL; BD Biosciences, San Jose, CA, USA) before cell seeding. For cardiotoxicity assays, 200,000 cells per well (6‐well format) were plated and allowed to form functional syncytia. Cells were maintained in RPMI medium supplemented with B‐27 plus (ThermoFisher Scientific, Waltham, MA, USA) and cultured for 7–10 days at 37 °C in 5% CO_2_ prior to functional measurements. For functional measurements, syncytia were loaded with the voltage‐sensitive dye FluVolt (ThermoFisher Scientific) to assess membrane potential of iPSC‐derived cardiomyocytes [39]. Ro 41‐0960 was prepared as a 10 mM stock solution in DMSO and diluted to working concentrations in Hank's balanced salt solution (SAFC Biosciences, Lenexa, KS, USA). Baseline recordings were obtained prior to treatment, followed by incubation with Ro 41‐0960 (0.5, 5, 50 µM) for 30 min. Optical mapping was performed using a high‐speed CCD camera (200 fps, 80 × 80 pixels) on a temperature‐controlled stage at 37 °C with LED illumination [19]. Conduction velocity and APD_90_ were quantified to assess electrophysiological effects [19, 40]. Data were acquired before and after treatment and analyzed using custom software [41, 42].

### 4.9 Statistical Analysis

All results are presented as mean ± standard error of the mean (SEM). Statistical significance was assessed using either a Student's *t*‐test or two‐way analysis of variance (ANOVA) followed by Dunnett's multiple‐comparison test, as appropriate. We used 95% confidence intervals around the differences between the groups for the post‐hoc test. Two‐sided *p‐*values were used, and an *α*‐level <0.05 was considered significant.

## Author Contributions

**Carlos Cruz‐Cortés**: investigation; methodology; formal analysis; writing – review and editing; visualization. **Jaroslava Šeflová**: investigation; methodology; funding acquisition; writing – review and editing. **Guadalupe Guerrero‐Serna**: investigation; methodology. **Eric N. Jimenez‐Vazquez**: investigation; methodology. **Justus Anumonwo**: resources; writing – review and editing. **L. Michel Espinoza‐Fonseca**: conceptualization; formal analysis; supervision; funding acquisition; writing – original draft; writing – review and editing.

## Funding

This work was supported by National Heart, Lung, and Blood Institute (R01HL176212).

## Conflicts of Interest

The authors declare no conflicts of interest.

## Supporting information

Supplementary Material

## Data Availability

The data that support the findings of this study are available from the corresponding author upon reasonable request.
