# Supplementary material for: Discovery of Ro 41‐0960, a Non‐Steroidal Inhibitor of the Na+/K+‐ATPase
Source: ChemMedChem. 2026 Aug 28;21(17):e70442. doi: 10.1002/cmdc.70442 (PMC13524838; doi:10.1002/cmdc.70442)
Supplement: Supplementary file 1 [file CMDC-21-e70442-s001.pdf]

Supporting Information

Discovery of Ro 41-0960, a Non-Steroidal Inhibitor of the Na^+/K^+-ATPase

Carlos Cruz-Cortés,[1,2] Jaroslava Šeflová,[3] Guadalupe Guerrero-Serna,[4] Eric N. Jimenez-Vazquez,[5] Justus Anumonwo,[1,2] and L. Michel Espinoza-Fonseca[1,2*]

[1]Department of Internal Medicine, Division of Cardiovascular Medicine, University of Michigan, Ann Arbor, MI 48109, USA

[2]Center for Arrhythmia Research, University of Michigan, Ann Arbor, MI 48109, USA

[3]Department of Cell and Molecular Physiology, Loyola University Chicago, Maywood, IL 60153, USA

[4]Frankel Cardiovascular Center, Department of Internal Medicine, Division of Cardiovascular Medicine, University of Michigan, Ann Arbor, MI 48109, USA

[5]Department of Pharmacology, University of Michigan, Ann Arbor, MI 48109, USA

Table S1. ATPase activities of SERCA and NKA preparations used in this study. Total ATPase activity was measured under standard assay conditions. Background ATPase activity corresponds to the activity remaining in the presence of saturating concentrations of thapsigargin for SERCA or ouabain for NKA. Specific ATPase activity was calculated as the difference between total and background ATPase activities, and specific activity (%) was calculated as the fraction of total activity that is sensitive to thapsigargin or ouabain. Values are presented as mean ± SEM.

Protein preparation	Total ATPase activity (µmol/mg/min)	Background ATPase activity (µmol/mg/min)	Specific ATPase activity (µmol/mg/min)	Specific activity (%)
SERCA microsomes	0.513 ± 0.017	0.117 ± 0.003	0.396 ± 0.019	77.2 ± 1.1
NKA microsomes	0.526 ± 0.027	0.312 ± 0.019	0.214 ± 0.011	40.6 ± 1.3
Purified NKA	1.455 ± 0.088	0.465 ± 0.050	0.990 ± 0.066	68.0 ± 2.6
